# Characterization of circRNA-Associated-ceRNA Networks Involved in the Pathogenesis of Postoperative Cognitive Dysfunction in Aging Mice

**DOI:** 10.3389/fnagi.2022.727805

**Published:** 2022-04-04

**Authors:** Meng-Xue Zhang, Jing-Run Lin, Shu-Ting Yang, Jun Zou, Yao Xue, Chen-Zhuo Feng, Lin Cao

**Affiliations:** ^1^Department of Anesthesiology, Sun Yat-sen Memorial Hospital, Sun Yat-sen University, Guangzhou, China; ^2^School of Basic Medical Sciences and Forensic Medicine, Hangzhou Medical College, Hangzhou, China

**Keywords:** postoperative cognitive dysfunction, aging, circular RNA, ceRNA network, RNA sequencing, neurocognitive function, hippocampus

## Abstract

Postoperative cognitive dysfunction (POCD) is a clinical entity associated with declined cognitive function following surgery. It occurs more frequently in elderly patients. Recent studies have shown that circRNA-associated-ceRNA networks, constructed based on interactions between circRNA-miRNA and miRNA-mRNA, provide key insight into the molecular mechanisms underlying the pathogenesis of several neurological diseases. However, the mechanism of POCD remains undetermined. In this study, laparotomies were performed under isoflurane anesthesia on young (2-month-old) and aging (17-month-old) male C57BL/6 mice. The results showed that the aging mice were more likely than the young mice to develop POCD. Subsequently, differentially expressed circRNAs, miRNAs, and mRNAs were characterized by RNA sequencing the hippocampi of young and aging mice under control and surgery conditions. Six circRNAs, 6 miRNAs, and 203 mRNAs were identified to construct the circRNA-associated-ceRNA network for the control condition, while 13 circRNAs, 8 miRNAs, and 189 mRNAs were used for the circRNA-associated-ceRNA network for the surgery condition. Further Gene Ontology (GO) and Kyoto Encyclopedia of Genes and Genomes (KEGG) pathway enrichment analysis of these two networks revealed that the circRNA-associated-ceRNA networks are involved in POCD pathogenesis though modulating the Wnt and VEGF signaling pathways, as well as neural processes associated with long-term synaptic depression and synaptic transmission. In particular, the mmu-miR-298-5P regulatory pathway identified in this study’s mouse model suggests that mm9_circ_009789- and mm9_circ_004229-associated-ceRNA networks as closely related to the occurrence of POCD through regulating PKC signaling pathway, neural cell apoptosis and glycolipid metabolism pathway. These findings provide possible insight into the role of the circRNA-associated-ceRNA networks, helping to unravel the complexity of the molecular pathogenesis of POCD.

## Introduction

Postoperative cognitive dysfunction (POCD) is a clinical entity that manifests as postoperative personality changes and impaired cognition, occurring mostly within a week or months after surgery (Safavynia and Goldstein, 2019; Urits et al., 2019). POCD is highly prevalent in elderly patients undergoing surgery, which induces prolonged hospital stays and increased mortality (Moller et al., 1998). However, its pathogenesis is unclear, and effective treatment drugs are lacking. Understanding the pathogenesis of POCD could help to find potential treatment target sites.

Previous studies have uncovered that the complicated pathogenesis of POCD is associated with various risk factors, and multiple mechanisms are involved (Canet et al., 2003; Pappa et al., 2017). Aging as one of the major risk factors, especially combined with surgery and anesthesia, has been proven to contribute to the development of POCD (Moller et al., 1998; Monk et al., 2008). Therefore, research on the effects of aging in POCD could provide new clues for exploring its pathogenesis.

With the development of RNA sequencing (RNA-seq) technology, studies have elucidated that a class of non-coding RNAs (ncRNAs) called circular RNAs (circRNAs), which are highly expressed in mammalian brains, is closely related to neurological functions (Danan et al., 2012; You et al., 2015). CircRNAs are covalently closed loop molecules without 5′ caps or 3′ poly-A tails formed *via* back-splicing of pre-mRNA (Danan et al., 2012). Acting as competing endogenous RNAs (ceRNA), circRNAs can combine with microRNAs (miRNAs) to remove the inhibitory effects on target mRNAs through microRNA response elements (MREs) (Salmena et al., 2011; Zhao et al., 2016; Li et al., 2018). The regulatory network formed by the interaction of circRNAs, miRNAs, and mRNAs is the circRNA-associated-ceRNA network (circRNA-miRNA-mRNA). Recently, increasing studies on circRNAs have revealed that circRNA-associated-ceRNA networks play a key role in the pathogenesis of Alzheimer’s disease (AD), stroke, and many other neurological diseases (Zhao et al., 2016; Lu et al., 2019; Wu et al., 2019), which provides a new clue for understanding the specific molecular interaction underlying POCD.

In this study, based on the POCD phenotype of the animal model of 17-month-old male C57BL/6 mice with laparotomies, the POCD-related differentially expressed (DE) circRNAs, miRNAs, and mRNAs were characterized by RNA-seq the hippocampi of 2- and 17-month-old male C57BL/6 mice under control and surgery conditions, and individual circRNA-associated-ceRNA networks were constructed. Then, elucidating the mmu-miR-298-5P regulatory pathway and comparing the differences between those networks with Gene Ontology (GO) and Kyoto Encyclopedia of Genes and Genomes (KEGG) pathway enrichment analysis, multiple mechanisms of POCD and the associated role of circRNA-associated-ceRNA networks were investigated.

## Materials and Methods

### Animal Groups

Young (2-month-old) and aging (17-month-old) male C57BL/6 mice were purchased from the Animal Center of Traditional Chinese Medicine, University of Guangzhou. The mice were housed in a climate-controlled room on a 12-h light/dark cycle and *ad libitum* access to food and water. The young and aging mice were randomly assigned to receive a laparotomy under isoflurane anesthesia or to the control treatment (control groups were not exposed to anesthesia or surgery). Young mice in the anesthesia/surgery group and aging mice in the control group had 8 mice subjected to behavioral tests. Young mice in the control group and aging mice in the anesthesia/surgery group had 9 mice subjected to behavioral tests. Each group had 3 mice euthanized for brain tissue preparation. The animal study was reviewed and approved by the Animal Ethics Committee of Sun Yat-sen University (SYSU-IACUC-2020-000458).

### Anesthesia and Surgery

Exploratory laparotomies were performed under isoflurane anesthesia. The mice were placed in an induction chamber anesthetized with 1.5–2.0% isoflurane and oxygen at 1 L/min for 30 min. Then the mice were placed on a mask connected to a vaporizer to maintain isoflurane (1.0–1.5%) in oxygen at 1 L/min during the surgery. The surgery region was cleaned with povidone iodine, and then an abdominal midline incision of about 1.5 cm was performed. A sterile cotton swab was inserted into the abdominal cavity to manipulate the viscera for 2 min. The small intestine was then exteriorized from the abdominal cavity, and rubbed gently between the thumb and index finger for 3 min. Finally, the muscle and skin were sutured with 4-0 chromic gut sutures. Anesthesia was ended immediately after the surgical procedure was done. The anesthesia and surgical procedure lasted approximately 70 min, and the surgical procedure was as sterile as possible. A rectal probe was used to monitor body temperature, which was maintained at 37° ± 0.5°C with a heating pad during surgery. After surgery, the mice were transferred into an incubator for 20 min and then returned to their enclosures. For the control groups, nothing was performed.

### Behavior Tests

#### Open Field Test

On Day 6 post-anesthesia/surgery, the mice were subjected to an open field test. The mice were placed in the center of a square arena ((50 × 50) cm^2^) and allowed to explore for 5 min. During the trial, the behavior of the mice was recorded by video, and their total distance moved and time spent in the center were calculated using Smart v3.0 software. Before each trial, the arena was cleaned with 75% ethanol.

#### Barnes Maze

Sixty minutes after the open field test, the mice were subjected to a Barnes maze test. The Barnes maze is a circular platform surrounded by 20 equally spaced holes, one of which was attached to a dark chamber, known as the target box. Before each trial, the platform and chamber were cleaned with 75% ethanol. The mice were followed with an aversive noise (85 dB) and bright light (200 W), all to encourage the mice to identify the target box. The mice were trained over a period of 4 days with 3 min per trial, and 2 trials per day within a 15-min interval. Then, the short-term retention and long-term retention were tested on Day 10 and Day 17 post-anesthesia/surgery, respectively. Only one trial was performed on each of those 2 days. Each trial was video recorded to capture the behavior of the mice finding the target box. The latency and routine to identify the target box were analyzed using Smart v3.0 software.

#### Fear-Conditioning Test

Twenty-four hours after the Barnes maze test, the mice were subjected to a fear conditioning test. Each mouse was placed in the test chamber, which was wiped down with 75% ethanol in a dark room and subjected to tone stimulus (2 kHz, 85 db) for 30 s and foot shock (0.7 mA) for 2 s. The tone-foot shock stimulation was repeated for three cycles within a 1- min inter-cycle interval. Then the mouse was placed in the same chamber 24 h later for 6 min without any stimulation. The time of contextual freezing behavior was recorded during the 6-min period. Two hours later, the mouse was placed in a different test chamber wiped with 1% acetic acid in a light room. After 3 min, the mouse was subjected to three tone stimulus cycles for 30 s within a 1-min inter-cycle interval. The tone freezing behavior was recorded during the 4.5-min period. The time of freezing behavior was counted by a Freeze Detector System (SD Instruments).

### Sample Preparation

The selected mice were deeply anesthetized with isoflurane and transcardially perfused with saline 12 h after surgery. The hippocampi were harvested immediately for RNA extraction.

### RNA Extraction, Purification, and RNA Sequencing

Total RNA was extracted from the hippocampi using an miRNeasy Mini Kit (Cat# 217004, Qiagen) according to the manufacturer’s instructions. RNA integrity was evaluated using an Agilent Bioanalyzer 2100 (Agilent Technologies, Santa Clara, CA, United States). Qualified RNA was further purified by an RNase-Free DNase Set (Cat#79254, Qiagen) and an RNAClean XP Kit (Cat#A63987, Beckman Coulter). The RNA libraries construction and sequencing on the Illumina HiSeq 2500 (Illumina, San Diego, CA, United States) were performed at Shanghai Biotechnology Corporation.

#### circRNA-seq

The isolated RNA used for circRNA-seq was treated with a RiboZero rRNA Removal Kit (Cat#RZG1224, Illumina) and RNase R (Cat#, Epicenter, RNR07250) for deleting rRNA and linear RNA. Next, the RNA samples were fragmented, and cDNA fragments were synthesized. After end reparation, purification and PCR amplification steps, the cDNA libraries were quantified and then sequenced with a Paired-End module.

#### miRNA-Seq

The isolated RNA was performed 3′ and 5′-end linker, reverse transcription and PCR amplification processes. Then the miRNA sequencing libraries were purified and quantified, and sequenced with a Single-End module.

#### mRNA-Seq

After deletion of rRNA, the isolated RNA used for mRNA-seq was fragmented. And the synthesized strand cDNA was performed an end repair and purified. Then enriched with PCR and the cDNA libraries were quantified and sequenced with a Paired-End module.

#### Differentially Expressed Genes Data Analysis

All Differentially Expressed Genes (DEGs) were identified using an edgeR package. The *P*-value was calculated by fisher’s test and the Benjamini–Hochberg correction was applied for multiple testing. For circRNA-seq, the fold-changes were estimated according to the SRPBM (back-splicing junction reads per billion mapping). For miRNA-seq, the fold-changes were estimated according to the TPM (transcripts per million reads). For mRNA-seq, the fold-changes were estimated according to the FPKM (fragments per kilobase of exon model per million mapped reads). The DEGs were selected using the following filter criteria: fold change ≥ 2, *P* ≤ 0.05.

#### CircRNA-Associated-ceRNA Network Construction

Based on the expression values of genes, a circRNA-associated-ceRNA network was established through linear regression model analysis and seed sequence matching methods. The specific methods are as follows: Predict DE miRNAs related to DE circRNAs and DE mRNAs through seed sequence matching analysis and then find circRNA-miRNA and miRNA-mRNA relationship pairs; take the circRNA-miRNA relationship pairs and the miRNA-mRNA relationship pairs to obtain the intersection miRNA; perform correlation coefficient cor_xy analysis on the circRNA and mRNA in the intersection to find a valid circRNA-mRNA relationship pair; use the gene expression value to establish the relationship value S between circRNA, miRNA, and mRNA to infer whether miRNA regulates circRNA and mRNA, and find out the relationship between the three; and lastly, the relationship value S is high, and there is a sequence matching association with miRNA. The ceRNA network diagram was produced using the OmicStudio tools at https://www.omicstudio.cn/tool/56.

### Real-Time qPCR Validation

CircRNAs and mRNAs were reverse transcribed using a PrimeScript RT Master Mix (Cat#RR036A, TaKaRa) and quantified using a TB Green Premix Ex Taq kit (Cat#RR820A, TaKaRa) according to the manufacturer’s instructions. miRNA was reverse transcribed and quantified using a miDETECT A TRACK miRNA qRT-PCR Starter Kit (Cat#R11068.5, RiboBio) according to the manufacturer’s instructions. The real-time qPCR reaction was performed in a Light Cycler 480 real-time PCR system (Roche Applied Science). The expression of circRNAs and mRNAs were normalized to b-actin, and the expression of miRNA was normalized to U6. The 2-ΔΔCt method was used to calculate the relative expression level. Three technological replicates were used to ensure the reliability of the analysis. The Uni-Reverse Primer and forward primer sequences for miRNA were obtained from RiboBio. The other primer sequences were designed and synthesized by RiboBio and are shown in Table 1.

**TABLE 1:** Primers Sequence for real-time qPCR.

Category	Sequences
mm9_circ_003736	Forward: AGCACAGCTCTGATTCCAGGAA
	Reverse: CCGCAGGAGACAATGCAAC
mm9_circ_009789	Forward: GAACCAGGACGAGCAGATTTGA
	Reverse: GGGTAGCACTCTTTCTCCTCCA
mm9_circ_004229	Forward: GAAGGTCACGGAGACCAAAGG
	Reverse: CTGACAGTCCTCTCCCGCAA
Zbtb4	Forward: CCGTAAATCTTGTTTCTGGCAC
	Reverse: CTCTCTTGTGGTTCCGCTGTA
Prkcb	Forward: CTGATAGCGGTACCTTCATCCC
	Reverse: TCCTTTGGGACCTGGCTAGAG
Syngap1	Forward: ATCTGCTCTTGGCTGGTCCC
	Reverse: CCCAACACACATGGCCTCCT
b-actin	Forward: TATGCTCTCCCTCACGCCATCC
	Reverse: GTCACGCACGATTTCCCTCTCAG
GAPDH	Forward: TGTGTCCGTCGTGGATCTGA
	Reverse: TTGCTGTTGAAGTCGCAGGAG

*Zbtb4, zinc finger and BTB domain containing 4; Prkcb, protein kinase C, beta; Syngap1, synaptic Ras GTPase activating protein 1.*

### Cell Culture and Transfection

Neuro-2A cell line was acquired for Procell (Wuhan, Hubei, China) and cultured in minimum essential medium (MEM) supplemented with 10% fetal bovine serum (FBS). Cells were kept at 37°C in air containing 5% CO_2_. The culture medium was replaced every 2 or 3 days. For capture of mmu-miR-298-5p-bound mRNA in the pull-down assay, cells were transfection with biotin-labeled control miRNA, mmu-miR-298-5p mimics. For analysis of mmu-miR-298-5p-bound circRNAs, mm9_circ_009789 and mm9_circ_004229 were cloned into pMS2 vector to generate plasmid pMS2- mm9_circ_009789 and pMS2-mm9_circ_004229 expressing MS2-tagged circRNAs. Then cells were co-transfected with plasmids pMS2, pMS2-mm9_circ_009789 or pMS2-mm9_circ_004229, and pMS2-GST that expresses MS2-binding protein and GST tag. Lipofectamine 2000 was used as transfection reagent according to the manufacturer’s instruction.

### RNA Pull-Down Assay

The biotinylated mmu-miR-298-5p mimic and a negative control miRNA were commercially synthesized (Ribobio, Guangzhou, China). The capture of mmu-miR-298-5p-bound mRNA in a pull-down assay was performed with biotinylated mmu-miR-298-5p mimics as described previously (Phatak et al., 2016; Awan et al., 2018). Briefly, 1 × 10^6^ Neuro-2A cells were transfected with biotin-labeled control miRNA or mmu-miR-298-5p mimics. Forty-eight hours later, cells were washed with ice-cold PBS and lysed in the lysis buffer containing 0.02 M Tris-HCl (pH 7.5), 0.1 M KCl, 5 mMMgCl_2_, 0.3% NP-40, 1X RNase inhibitor and 1X protease inhibitor. Lysates were cleared off by centrifugation at 13,000 rpm for 10 min and incubated with streptavidin-coupled magnetic beads at 4°C overnight for pull-down. MS2-TRAP assay was performed to capture circRNA associated mmu-miR-298-5p (Yoon et al., 2012). MS2-tagged circRNAs and that associated miRNA was pulled down by anti-GST magnetic beads. RNA was isolated with TRIzol reagent for the subsequent assessment of the mRNA and miRNA. The relative enrichments of RNAs were normalized by the levels of GAPDH in each reaction.

### Gene Annotation and Enrichment Analysis

Metascape^1^ was used to perform the Gene annotation and enrichment analysis (Zhou et al., 2019). The analysis included the following steps: A. ID conversion: Convert input gene identifiers into M. musculus Entrez Gene IDs. B. Gene annotation: KEGG pathway analysis^2^ and GO analysis^3^ were performed. Extract annotation columns for the gene list using GO molecular functions, GO biological processes, GO cellular components and KEGG pathways. C. Membership analysis: Flag genes that fall under interest terms. The *P*-value indicates whether the membership is statistically enriched in the input gene list and is calculated based on hypergeometric test and Benjamini-Hochberg correction for multiple testing. *P* < 0.05 is considered statistically significant. D. Enrichment analysis: Terms with a *P*-value < 0.05, a minimum count of 3, and an enrichment factor > 1.5 are used for filtering the significant terms. The significant terms have been converted as a network, where terms are represented by circle nodes and connected by edges with a similarity > 0.3. Each node is represented as a pie chart. The color of each pie sector represents different gene lists, and the area of each pie shows the positive correlation to the number of genes related to that term, as derived from the input gene list. The network is visualized using Cytoscape software (version 3.1.2).

### Statistical Analysis

Results were expressed as the mean ± SEM. The data from the Barnes maze test training sessions for each group was tested by one-way repeated measures analysis of variance followed by Tukey’s test. The data from the real-time qPCR and RNA pull-down were tested by a Student’s *t*-test. The other data were tested by two-way analysis of variance followed by Tukey’s test. P_surg_, P_age_, and P_int_ presented the *P*-values of anesthesia/surgery, age, and interaction of factors, respectively. *P* < 0.05 was considered statistically significant. All statistical analyses were performed with GraphPad Prism 7.0 software.

## Results

### Anesthesia/Surgery Caused Age-Dependent Memory Impairments in Mice

**FIGURE 1:**
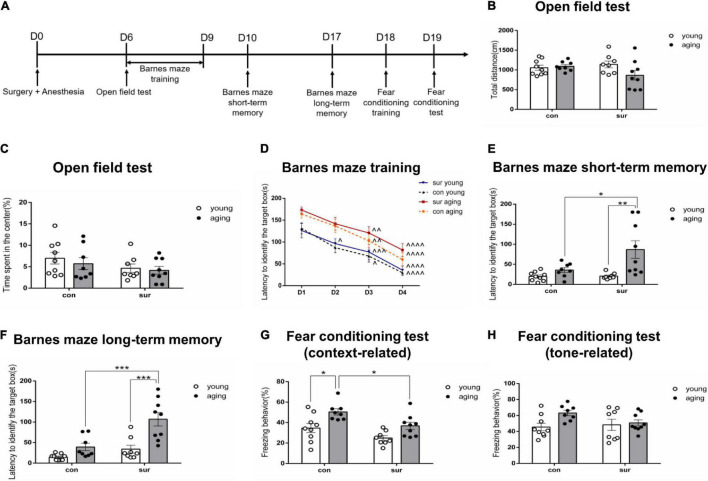
Anesthesia/surgery caused age-dependent memory impairments in mice. **(A)** Timeline for behavioral tests. **(B,C)** Total distance moved and time spent in the center were recorded during the open field test. **(D–F)** Latency to identify the target box was recorded in the Barnes maze. **(D)** Barnes maze training sessions. **(E)** Barnes maze short-term memory test. **(F)** Barnes maze long-term memory test. **(G,H)** The time of contextual and tone freezing behavior was recorded during the fear conditioning test. ^∧^*p* < 0.05, ^∧∧^*p* < 0.01, ^∧∧∧^*p* < 0.001,^∧∧∧∧^*p* < 0.0001 compared with the corresponding data on Day 1. *P < 0.05, **P < 0.01 and ***P < 0.001 for pairwise comparisons. All data are presented as mean ± SEM (*n* = 8 for young mice in the anesthesia/surgery group and aging mice in the control group. *n* = 9 for young mice in the control group and aging mice in the anesthesia/surgery group).

Given that POCD is more likely to occur among the elderly, the behavioral performance of both the young and aging mice was tested after either anesthesia/surgery or the control treatment. Behavioral tests were performed starting on Day 6 post-anesthesia/surgery to avoid the influence of any pain or discomfort caused by surgery (Figure 1A). On Day 6 post-anesthesia/surgery, the open field test was performed first to test the locomotor activity and anxiety-like behavior of the mice. The results showed that anesthesia/surgery did not change the total movement distance [P_surg_ = 0.4091, F_surg(1, 30)_ = 0.7010; P_age_ = 0.1735, F_age(1, 30)_ = 1.944; P_int_ = 0.0789, F_*int*(1, 30)_ = 3.310] or time spent in the center [P_surg_ = 0.1059, F_surg(1, 30)_ = 2.780; P_age_ = 0.4604, F_age(1, 30)_ = 0.5592; P_int_ = 0.7425, F_int(1, 30)_ = 0.1099] as compared to the control condition between young and aging mice (Figures 1B,C).

For the Barnes maze, the mice were trained daily from Days 6 through 9 post-anesthesia/surgery. Then, the tests were conducted on Days 10 and 17 in order to investigate hippocampus-dependent short-term and long-term memory (Barnes, 1979; Raber et al., 2004). The results showed that training shortened the time to identify the target box in each group (Figure 1D). Analysis of the short-term memory test showed that anesthesia/surgery significantly prolonged the time to identify the target box in aging mice as compared to those in the control group. [P_surg_ = 0.0422, F_surg(1, 30)_ = 4.502; P_age_ = 0.0021, F_*age*(1, 30)_ = 11.29; P_int_ = 0.0476, F_int(1, 30)_ = 4.266] (Figure 1E). Similar change was observed in the long-term memory test [P_surg_ = 0.0004, F_surg(1, 30)_ = 16.06; P_age_ < 0.0001, F_age(1, 30)_ = 20.24; P_int_ = 0.0333, F_int(1, 30)_ = 4.979] (Figure 1F).

To further confirm the effect of anesthesia/surgery on aging mice, a fear conditioning test was used to determine associative memory on Day 18 post-anesthesia/surgery (Phillips and LeDoux, 1992; Ciocchi et al., 2010). The results showed that anesthesia/surgery decreased freezing behavior in aging mice, but not in young mice (*P* = 0.0498, Tukey’s multiple comparisons test). The significant difference between young and aging mice under control conditions (*P* = 0.0180, Tukey’s multiple comparisons test) disappeared when the mice were under anesthesia/surgery conditions in a context-related test, although the interaction of anesthesia/surgery and age was not significant [P_surg_ = 0.0025, F_surg(1, 30)_ = 10.90; P_age_ = 0.0005, F_age(1, 30)_ = 15.34; P_int_ = 0.5892, F_int(1, 30)_ = 0.2979] (Figure 1G). Analysis results did not show any differences among the four groups in the tone-related test, which requires proper function of the amygdala (Dulka et al., 2021). [P_surg_ = 0.3238, F_surg(1, 30)_ = 1.006; P_age_ = 0.0464, F_age(1, 30)_ = 4.315; P_int_ = 0.1269, F_*int*(1, 30)_ = 2.465] (Figure 1H).

### Anesthesia/Surgery Caused Age-Dependent Changes in the Hippocampal Transcriptome

To understand the molecular regulatory mechanism of anesthesia/surgery-induced, age-dependent memory decline in mice, the DE circRNAs, miRNAs, and mRNAs of the hippocampi were further screened using RNA-seq to compare young and aging mice under control and anesthesia/surgery conditions. To obtain high-quality reads, the low-quality bases and reads were filtered out. For circRNA-seq, *via* mapping to the mouse reference sequence (GRCm38.p3, NCBI) by BWA-MEM (Li, 2013), the total number of clean reads in young and aging mice under the control condition was 201,156,959 (99.41%) and 201,658,617 (99.64%), respectively. Under the anesthesia/surgery condition, there were 207,612,958 (99.40%) and 201,459,368 (99.67%) clean reads in young and aging mice, respectively. For miRNA-seq, a total of 67,358,177 (97.91%) and 69,655,422 (97.87%) clean reads were mapped to the mouse reference sequence (GRCm38.p3, NCBI) by Bowtie in young and aging mice under control conditions (Langmead et al., 2009), while there were 70,533,956 (97.79%) and 68,178,536 (97.87%) clean reads in young and aging mice under anesthesia/surgery conditions. For mRNA-seq, 217,461,629 (97.90%) and 200,020,472 (98.21%) clean reads in young and aging mice under control conditions were mapped to the mouse reference genome (GRCm38.p3, NCBI) by Hisat2 (version:2.0.4) (Kim et al., 2015). Meanwhile, there were 201,470,337 (98.19%) and 208,430,151 (98.13%) clean reads in young and aging mice under anesthesia/surgery conditions. The RNA-seq data were uploaded to the SRA database with the accession number: SRP323354. Collectively, 106,293 circRNAs (1,451 known and 104,842 novel), 1,235 miRNAs (1,204 known and 31 novel), and 64,338 protein coding transcripts were detected in our study samples. The known circRNAs, miRNAs, and mRNAs were used for subsequent analysis.

**FIGURE 2:**
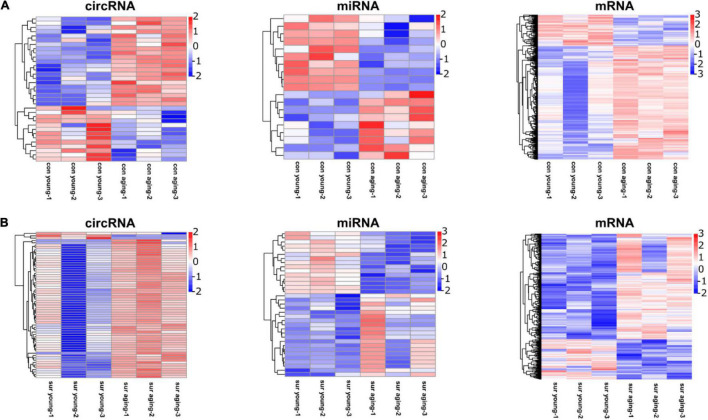
Anesthesia/surgery caused age-dependent changes in the hippocampal transcriptome. **(A)** Heatmap representing DE circRNAs, miRNAs, mRNAs in hippocampus between young and aging mice under control conditions. **(B)** Heatmap representing DE circRNAs, miRNAs, mRNAs in hippocampus at 12 h post-anesthesia/surgery between young and aging mice under anesthesia/surgery conditions. DE genes were filtered according to fold change ≥ 2, *P* ≤ 0.05. *n* = 3 per group. Pink color represents upregulated expression. Blue color represents downregulated expression.

The DE circRNAs, miRNAs, and mRNAs between young and aging mice under different experimental conditions were identified by the following parameters: fold change ≥ 2 and *P* ≤ 0.05. Compared to the gene profiling of young mice, 34 DE circRNAs (21 upregulated; 13 downregulated) (Supplementary Table 1), 19 miRNAs (9 upregulated; 10 downregulated) (Supplementary Table 2), and 576 mRNAs (453 upregulated, 123 downregulated) (Supplementary Table 3) were identified in the hippocampi of aging mice under control conditions (Figure 2A), and 59 DE circRNAs (56 upregulated, 3 downregulated) (Supplementary Table 4), 35 miRNAs (20 upregulated, 15 downregulated) (Supplementary Table 5), and 445 mRNAs (322 upregulated, 123 downregulated) (Supplementary Table 6) were identified in the hippocampi of aging mice under anesthesia/surgery conditions (Figure 2B).

### Anesthesia/Surgery Caused Age-Dependent Changes in CircRNA-Associated-ceRNA Networks

**FIGURE 3:**
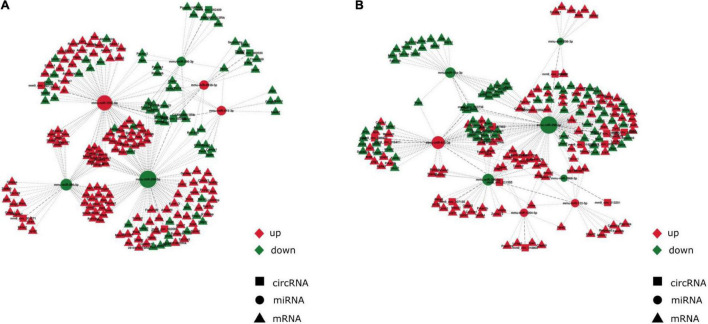
Anesthesia/surgery caused age-dependent changes in circRNA-associated-ceRNA networks. The ceRNA networks are constructed by the interactions of circRNA-miRNA and miRNA-mRNA **(A)** CircRNA-associated-ceRNA network under control conditions. **(B)** CircRNA-associated-ceRNA network under anesthesia/surgery conditions. Red color represents upregulated expression. Green color represents downregulated expression.

Two circRNA-associated-ceRNA networks were constructed under control and anesthesia/surgery conditions, based on the specifically identified DE circRNAs, miRNAs, and mRNAs (Figures 3A,B, respectively). The control network was constructed by 6 circRNAs, 6 miRNAs, and 203 mRNAs, and their binding sites are shown in Table 2; while the anesthesia/surgery network was constructed by 13 circRNAs, 8 miRNAs, and 189 mRNAs, and the predicted binding sites of miRNA are shown in Table 3. Detailed ceRNA networks are listed in Supplementary Tables 7, 8. Changes were evident in the networks related to DE circRNAs induced by anesthesia/surgery. Notably, mmu-miR-298-5P, whose expression was significantly lower in aging mice under both experimental conditions, interacted with circRNAs to regulate mRNA expression. The circRNA mm9_circ_003736, mm9_circ_009789, mm9_circ_004229, and mm9_circ_008009, whose expressions were changed significantly in aging mice that underwent anesthesia/surgery, have the same target miRNA, mmu-miR-298-5P. The associated ceRNA networks of the four top DE circRNAs were selected for subsequent enrichment analysis.

**TABLE 2:** DE circRNAs and miRNAs in ceRNA networks under control conditions.

CircRNA	log_2_^Fold Change^	*P*-value	Up/down regulation	circRNA-miRNA
mm9_circ_001571	1.55	0.0197	Up	mmu-miR-296-5p
mm9_circ_002005	1.27	0.0010	Up	mmu-miR-298-5p
mm9_circ_002179	1.25	0.0024	Up	mmu-miR-7056-5p
mm9_circ_002489	–1.11	0.0196	Down	mmu-miR-296-3p
mm9_circ_000550	–1.66	0.0025	Down	mmu-miR-6938-3p
mm9_circ_003736	–2.71	6.88E-27	Down	mmu-miR-298-5p, mmu-miR-615-3p, mmu-miR-6938-3p, mmu-miR-7056-5p

*Up/downregulation represents the circRNA upregulated or downregulated in aging mice under control conditions. circRNA-mRNA is the miRNA that was predicted to bind circRNA through seed sequence matching.*

**TABLE 3:** DE circRNAs and miRNAs in ceRNA networks under anesthesia/surgery conditions.

circRNA	log_2_^Fold Change^	*P*-value	Up/down regulation	circRNA-miRNA
mm9_circ_006949	1.08	0.0449	Up	mmu-miR-615-3p
mm9_circ_007155	1.24	0.0497	Up	mmu-miR-296-5p
mm9_circ_010522	1.30	0.0466	Up	mmu-miR-298-5p
mm9_circ_013251	1.28	0.0489	Up	mmu-miR-1966-3p
mm9_circ_014796	1.19	0.0350	Up	mmu-miR-298-5p
mm9_circ_016411	1.21	0.0216	Up	mmu-miR-615-3p
mm9_circ_018802	1.24	0.0459	Up	mmu-miR-1264-5p
mm9_circ_009789	1.98	0.0003	Up	mmu-miR-298-5p
mm9_circ_008009	1.48	0.0316	Up	mmu-miR-298-5p
mm9_circ_004229	1.52	0.0109	Up	mmu-miR-298-3p, mmu-miR-298-5p
mm9_circ_007503	1.31	0.0042	Up	mmu-miR-298-5p, mmu-miR-615-3p
mm9_circ_011555	1.50	0.0149	Up	mmu-miR-122-5p, mmu-miR-615-3p
mm9_circ_003736	–2.46	2.93E-18	Down	mmu-miR-18a-3p, mmu-miR-298-5p mmu-miR-615-3p

*Up/downregulation represents the circRNA upregulated or downregulated in aging mice under anesthesia/surgery conditions. circRNA-mRNA is the miRNA that was predicted to bind circRNA through seed sequence matching.*

### Real-Time qPCR and RNA Pull-Down Validated CircRNA-Associated-ceRNA Networks

**FIGURE 4:**
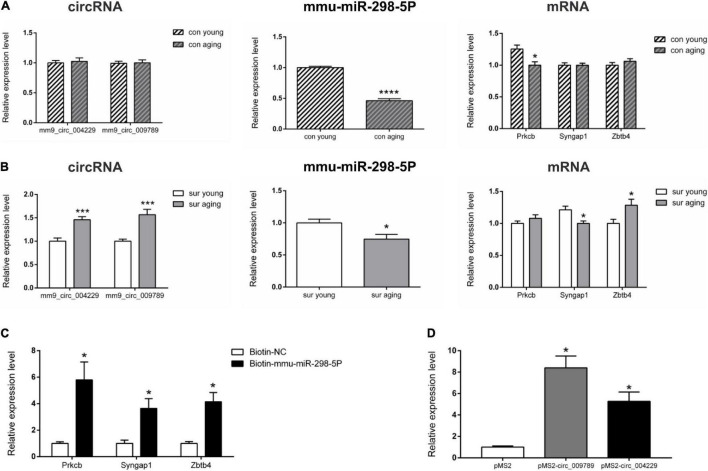
Real-time qPCR validation and RNA pull down of circRNA-associated-ceRNA networks. **(A,B)** Real-time qPCR to analysis the expression of mm9_circ_004229, mm9_circ_009789, mmu-miR-298-5P, Prkcb, Syngap1 and Zbtb4 in young and aging mice under control and anesthesia/surgery conditions (*n* = 6). **(C)** RNA pull-down assay to analysis mmu-miR-298-5P targets genes Prkcb, Syngap1 and Zbtb4 in Neuro-2A cell. **(D)** RNA pull-down assay to analysis mm9_circ_004229/mm9_circ_009789 associated mmu-miR-298-5P in Neuro-2A cell. *p < 0.05, ***p < 0.001, ****p < 0.0001. All data are presented as mean ± SEM.

Real-time qPCR was carried out before enrichment analysis to confirm the DE circRNAs, miRNAs, and mRNAs (Figures 4A,B). Compared to the expression patterns of circRNAs between control young and aging mice, the differential expression patterns of circRNA mm9_circ_009789 and mm9_circ_004229-associated-networks were validated under control and anesthesia/surgery conditions, respectively. Those transcripts were highly correlated with dysregulation in the anesthesia/surgery-related networks. The expression levels of mm9_circ_009789 and mm9_circ_004229 which were similar in control young and aging mice were higher in aging mice than young mice under anesthesia/surgery conditions. It was also noted that mmu-miR-298-5p, the target of both circRNAs, was downregulated in aging mice under both conditions. It was then confirmed that the targets of mmu-miR-298-5p, included the transcripts of the genes Prkcb, Syngap1, and Zbtb4, were differentially expressed under different conditions. The transcript of the gene Prkcb was only downregulated in the aging mice under control conditions, while the transcript of the gene Syngap1 was only downregulated in the aging mice under anesthesia/surgery conditions. In addition, the upregulated levels of the gene Zbtb4 were confirmed in the aging mice only under anesthesia/surgery conditions. Thus, the qRT-PCR results were shown to be consistent with the sequencing data.

Further, RNA pull-down assay results showed that the transcripts of the genes Prkcb, Syngap1, and Zbtb4 were pulled down by biotin-labeled mmu-miR-298-5p probe, indicating that mmu-miR-298-5p might directly interacted with those three gene (Figure 4C). It was also reported circRNAs can regulate gene expression *via* acting as a sponge to impact miRNA function. To test whether circRNA mm9_circ_009789 and mm9_circ_004229 associated with mmu-miR-298-5p, we performed MS2 TRAP assay. The results showed that mmu-miR-298-5p was pulled down in the cells transfected with pMS2- mm9_circ_009789 and pMS2- mm9_circ_004229, while not in the cells transfected with pMS2, which indicated that mm9_circ_009789 and mm9_circ_004229 could bind to mmu-miR-298-5p (Figure 4D).

### Gene Annotation and Enrichment of Target Gene mRNAs in ceRNA Networks

**FIGURE 5:**
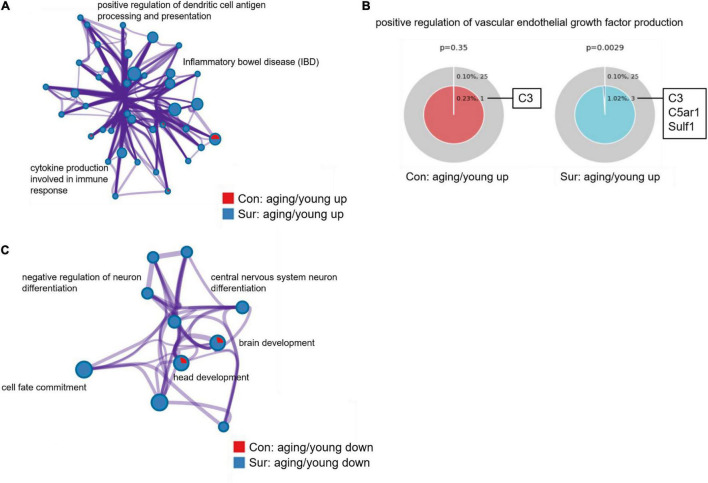
Gene annotation and enrichment of dysregulated mRNAs. **(A,B)** The enrichment analysis of upregulated mRNAs between young and aging mice under control (red) and anesthesia/surgery (blue) conditions. **(A)** Enrichment network constructed by enriched terms. **(B)** Enriched GO term: “positive regulation of vascular endothelial growth factor production” (GO:0010575). The term related mRNAs are as shown in the figure. *P* < 0.05 is considered as statistically significant. **(C)** The enrichment analysis of downregulated mRNAs between young and aging mice under control (red) and anesthesia/surgery (blue) conditions. And enrichment network constructed by enriched terms.

Before enrichment analysis of target mRNAs in ceRNA networks, an enrichment analysis of all dysregulated mRNAs was performed to validate the results of the mRNA screening, including some mRNAs that were not in the ceRNA networks. Many inflammatory processes such as positive regulation of dendritic cell antigen processing and presentation (GO:0002606), inflammatory bowel disease (ko05321) and cytokine production involved in immune response (GO:0002367) were found to be only enriched in the upregulated mRNAs of aging mice induced by anesthesia/surgery (Figure 5A). In addition, as shown in Figure 5B, mRNAs related to the positive regulation of vascular endothelial growth factor production (GO:0010575) such as the mRNAs C5ar1 and Sulf1 were only overexpressed in the aging mice that underwent anesthesia/surgery. Conversely, downregulated mRNAs of aging mice that underwent anesthesia/surgery were enriched in the central nervous system neuron differentiation (GO:0021953), negative regulation of neuron differentiation (GO:0045665) and other processes related to the development of the nervous system (Figure 5C).

**FIGURE 6:**
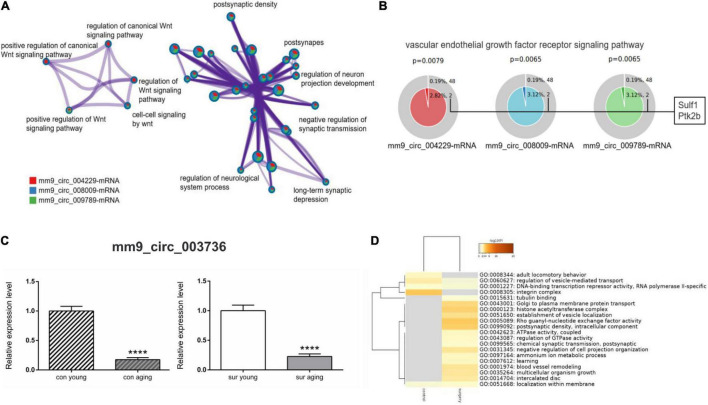
Gene annotation and enrichment of target mRNAs in ceRNA networks. **(A,B)** The enrichment analysis of target mRNAs derived from mm9_circ_004229/mm9_circ_008009/mm9_circ_009789-associated-ceRNA networks [mm9_circ_004229-mRNA (red), mm9_circ_008009-mRNA (blue) and mm9_circ_009789-mRNA (green)]. **(A)** Enrichment network constructed by enriched terms. **(B)** Enriched GO term: “Vascular endothelial growth factor receptor signaling pathway” (GO:0048010). The term related mRNAs are as shown in the figure. *P* < 0.05 is considered as statistically significant. **(C,D)** The analysis of mm9_circ_003736-associated-ceRNA network. **(C)** Real-time qPCR to analysis the expression of mm9_circ_003736 in young and aging mice under control and anesthesia/surgery conditions. ****p < 0.0001. All data are presented as mean ± SEM (*n* = 6). **(D)** The enrichment analysis of target mRNAs derived from mm9_circ_003736-associated-ceRNA networks under control and anesthesia/surgery conditions. Heatmap representing the differences between control and anesthesia/surgery conditions, colored by *p*-values, as shown in the legend above. The darker the color, the more statistically significant the term is. Gray indicates no enrichment in the corresponding term, *P* > 0.05.

Next, enrichment analysis was performed comparing mRNAs from three ceRNA networks (mm9_circ_009789, mm9_circ_004229, and mm9_circ_008009-mRNA) between control mice and anesthesia/surgery mice. Those circRNAs were all upregulated significantly in the aging mice induced by anesthesia/surgery. The enrichment analysis showed that mRNAs involved in those ceRNA networks were enriched in processes related to the Wnt signaling pathway such as positive regulation of the Wnt signaling pathway (GO:0030177) and cell-cell signaling by Wnt (GO:0198738), and also some processes related to neurological dysfunctions such as negative regulation of synaptic transmission (GO:0050805) and long-term synaptic depression (GO:0060292) (Figure 6A). In addition, Sulf1 and Ptk2b, as predicted downstream mRNAs upregulated by the top 3 upregulated circRNAs, were found to be related to the vascular endothelial growth factor receptor signaling pathways (GO:0048010) (Figure 6B). The data from the enrichment analysis of mm9_circ_003736 networks was also compared between control and anesthesia/surgery conditions, although the expression pattern of mm9_circ_003736 was similar (Figures 6C,D). The mRNAs in the mm9_circ_003736-associated-ceRNA network associated with anesthesia/surgery were enriched in the learning (GO:0007612), chemical synaptic transmission, postsynaptic (GO:0099565) and postsynaptic density, and intracellular component (GO:0099092).

## Discussion

This study focused on the effect of advanced age and anesthesia/surgery on POCD, since prior research has found that elderly patients are more likely to develop POCD after surgery than young patients (Moller et al., 1998; Monk et al., 2008). Given that anesthesia/surgery-induced gene response in the brain is age-dependent, however, how it results in the development of POCD remains unclear. To identify the potential circRNAs functioning as ceRNA, which regulate gene expression through shared miRNA (Feng et al., 2019; Yu et al., 2019), a POCD mice model was used to characterize the differences of hippocampal transcriptome responses to anesthesia/surgery between young and aging mice under control and anesthesia/surgery conditions through RNA-seq, and then the circRNA-associated-ceRNA networks involved in the pathogenesis of POCD were explored.

Modeling POCD-like symptoms in mice is difficult, as no animal model can quite mimic all of the POCD symptoms found in humans. This study used exploratory laparotomy, which is one of the most widely used methods for inducing POCD-like symptoms in mice (Barrientos et al., 2012; Kawano et al., 2015; Qiu et al., 2016). POCD is characterized by long-term memory impairments, which indicates dysregulation of hippocampal function in patients (Yonelinas, 2013; Tatu and Vuillier, 2014). The Barnes maze and contextual fear-conditioning test showed that anesthesia/surgery impaired long-term hippocampus-dependent memory in aging mice but not in young mice. These results are consistent with other studies (Culley et al., 2003; Barrientos et al., 2012), which indicates that the mouse model from this study could help to further investigate age-related POCD neuropathogenesis.

Dysregulated circRNA-associated-ceRNA networks were identified in current study by comparing the different expression patterns of circRNAs, miRNAs, and mRNAs between young and aging mice under control and anesthesia/surgery conditions. Particularly, the mmu-miR-298-5P regulatory pathway showed closely related to the POCD pathogenesis. The upregulation of mm9_circ_009789 and mm9_circ_004229 may act as a sponge of mmu-miR-298-5P to regulate the expressions of Prkcb, Zbtb4 and Syngap1 in mice hippocampi. The protein PRKCB encoded by Prkcb is a member of protein kinase C (PKC) family which was reported as a target of volatile anesthetics and overactivated may induce synaptic impairments and working memory dysfunction (Moe et al., 2003; Tagawa et al., 2015). ZBTB4 belongs to zinc finger protein family which is a DNA-binding protein and repress neuronal gene transcription (Filion et al., 2006). It was suggested to promote cell apoptosis and repress glycolipid metabolism pathway through inhibiting the expression of hexokinase 2 and ATP citrate lyase, the key enzymes related to glycolipid metabolism (Dong et al., 2021). Given that the abnormal glycolipid metabolism was associated with cognitive dysfunction (Arend et al., 2018; Qiu et al., 2022), the changes in the expression of mm9_circ_009789, mm9_circ_004229, Prkcb and Zbtb4 in our POCD model suggests that the PKC signaling pathway, neural cell apoptosis and glycolipid metabolism pathway may be regulated by mm9_circ_009789 and mm9_circ_004229-associated-ceRNA networks and involved in age-related POCD neuropathogenesis. Another target of mmu-miR-298-5P, Syngap1, was downregulated instead of upregulated in our POCD model, which may because it was powerfully affected by other ways of gene expression regulation rather than mmu-miR-298-5P. Syngap1 plays critical roles in regulate synaptic plasticity and neuronal homeostasis (Jeyabalan and Clement, 2016). Syngap1 mutation or haploinsufficiency causes a severe neurodevelopmental disorder with a phenotype of cognitive impairment (Kilinc et al., 2018; Nakajima et al., 2019). The lower expression of Syngap1 in aging mice with anesthesia/surgery in our study coincided with their long-term memory impairments.

For enrichment analysis on mRNAs derived from mm9_circ_009789- and mm9_circ_004229-associated-ceRNA networks, enrichment analysis was first performed on all dysregulated mRNAs to validate the results of the mRNA screening, and then the dysregulated mRNAs related to the networks and their associated biological functions were investigated. Enrichment analysis showed that compared to the age-dependent differential expression of mRNAs, the dysregulated mRNAs induced by anesthesia/surgery were enriched in several inflammation-related processes. This is consistent with previous studies that hypothesized that neuroinflammation may be the primary mechanism of POCD (Cao et al., 2010; Terrando et al., 2010). The upregulation levels of mm9_circ_009789 and mm9_circ_004229 may increase the expression of mRNAs related to positive regulation of the Wnt signaling pathway (GO:0030177), which has also been associated with neuroinflammation through enhancing microglial activation to modulate inflammatory responses (Marchetti and Pluchino, 2013; Steenwinckel et al., 2019; Yao et al., 2019). The findings in this study not only confirm that neuroinflammation is involved in the pathogenesis of POCD, but also suggest that circRNA-associated-ceRNA networks may promote neuroinflammation in POCD through regulating the Wnt signaling pathway.

Another finding in this study was that anesthesia/surgery induced dysregulated expression of Sulf1 in aging mice, which is involved in the positive regulation of vascular endothelial growth factor (VEGF) production (GO:0010575). The Sulf1 mRNA encoded an extracellular heparan sulfate endosulfatase, which is expressed widely in the rodent nervous system (Nagamine et al., 2005; Joy et al., 2015), and can regulate the VEGF signaling pathway and participate in the developing nervous system (Kalus et al., 2009; Gorsi et al., 2014). The level of Sulf1 mRNA in aging mice after anesthesia/surgery was predicted to be upregulated by mm9_circ_009789 and mm9_circ_004229 through competitively binding with mmu-miR-298-5P. Thus, there was an assumption that VEGF may play an important role in the pathogenesis of POCD and that circRNA-associated-ceRNA networks may be involved in POCD mechanisms through the triggering of VEGF signaling pathways. In addition, although the expression pattern of mm9_circ_003736 was not changed by anesthesia/surgery, mRNAs related to learning, synaptic transmission, and postsynaptic density may be regulated in the mm9_circ_003736-associated-ceRNA network under anesthesia/surgery conditions, which suggests that the age-dependent expression of mm9_circ_003736 may also be related to the occurrence of POCD.

There were some limitations in the study. Firstly, this study was limited to male mice since potential influence of estrous cycle on cognition in female mice should be avoided. However, similar study in female mice need to be conducted. Secondly, considering that surgery and anesthesia are always conducted simultaneously in a clinical setting, and that it is difficult to ascertain precisely how anesthesia and surgery each contribute to POCD, an individual anesthesia group was not included in this study. In addition, it is known that the behavioral test itself is a kind of stimulation for mice, which may induce the expression of dysregulated genes (Hansen et al., 2013). To avoid this effect, the mice used for RNA-seq were not subjected to behavioral tests. Although RNA-seq was done on the basis of behavioral confirmation that the surgical model can be used in studying POCD, additional replication of the RNA-seq data is necessary. Finally, and most importantly, future studies will be required to confirm the circRNAs-associated-ceRNA networks identified in this research.

## Conclusion

In conclusion, this study confirmed that aging mice are more likely than young mice to develop POCD. Through enrichment analysis of POCD-related changes in the hippocampal transcriptome of aging mice, it was discovered that in the POCD phenotype of aging male mice, POCD pathogenesis is complex, involving neuroinflammation, neurodevelopment, and other aspects. circRNA-associated-ceRNA networks may participate in the pathogenesis of POCD through regulating various pathways such as PKC signaling pathway, Wnt signaling pathway, VEGF signaling pathway and glycolipid metabolism pathway, as well as some neural functional processes, including long-term synaptic depression, synaptic transmission and neural cell apoptosis.

## Data Availability Statement

The datasets presented in this study can be found in online repositories. The names of the repository/repositories and accession number(s) can be found in the article/Supplementary Material.

## Ethics Statement

The animal study was reviewed and approved by the Animal Ethics Committee of Sun Yat-sen University.

## Author Contributions

M-XZ, J-RL, C-ZF, and LC contributed to conception and design of the study. M-XZ and J-RL performed the experiments and analyzed the data. S-TY, JZ, and YX contributed to materials and analysis tools. M-XZ wrote the first draft of the manuscript. All authors contributed to manuscript revision, read, and approved the submitted version.

## Conflict of Interest

The authors declare that the research was conducted in the absence of any commercial or financial relationships that could be construed as a potential conflict of interest.

## Publisher’s Note

All claims expressed in this article are solely those of the authors and do not necessarily represent those of their affiliated organizations, or those of the publisher, the editors and the reviewers. Any product that may be evaluated in this article, or claim that may be made by its manufacturer, is not guaranteed or endorsed by the publisher.

## Footnotes

1
http://metascape.org

2
https://www.genome.jp/kegg/

3
http://geneontology.org/
